# Tetanus Successfully Treated

**Published:** 1853-09

**Authors:** J. C. Billingslea

**Affiliations:** Tuscaloosa Co., Alabama


### SELECTIONS.

From the Southern Med. and Surg Journal.

*Tetanus, successfully treated.* By J. C. Billingslea, M.D., of Tuscaloosa Co., Alabama.

*My Dear Sir*—Below you will find an account of a case of tetanus which fell under my care recently. If you think it worthy of a place in your journal you can publish it.

April 27th, 1853. Was called to see a young negro man, aged 18 years, the property of Mr. F. I was informed that, four days previous to this, he had been thrown from a mule, and after the fall, he complained of "the small of his back and breast hurting him." I found the boy sitting up at 12 M. He had considerable fever; pulse 120 per minute, and resisting; tongue foul; skin very dry; not much ability on the part of the patient to move his lower extremities; considerable tenderness of the spine about the last dorsal and first lumbar vertebræ; his chest somewhat tumefied and painful to touch, just above the epigastrium. He said the mule had trodden on him. In addition to these, my attention was called to his left foot, the great toe of which had been frostbitten nearly eighteen months before, but was thought by his master to be *well*, until he received the fall. The toe was much swollen, and had a cavity in the end, about a quarter of a inch in diameter, which extended to the bone and was filled with pus. The whole foot was very much swollen and tender. He did not know whether it was hurt in the fall or not, but said it did not commence swelling till after the fall. He was robust and strong. I bled him from a large orifice, but took only a small quantity before a sensible impression was made on his pulse, and he expressed himself as free of pain, and much better. I then applied cups over the tender portion of the spine, and afterwards sinapisms to the same—then ordered him the following: ℞. Calomel, Dover's powders and sulph. quinine, each 12 grs., divided into six powders—one to be taken every two hours. Tinct. of iodine to be applied to the foot, followed by a cold poultice. He had no tetanic symptoms that I discovered at this visit, although he said the pain in his chest came on by paroxysms.

28th, 11 o'clock, P. M. Was called in haste to him. I found him with pulse 120 per minute; tongue moist and cleaning; slight diaphoresis; and whenever touched, or spoken to, he would be thrown into violent opisthotonos, and during each spasm would complain of his chest. I think there was not more than three minutes of repose between the spasms. I was informed that he had been in this condition all day—his medicine had not acted on his bowels; fortunately, he could open his mouth about half an inch, and deglutition was not impaired. I gave him an enema of castor oil and spts. of turpentine, which I repeated frequently, until I procured a full evacuation of his bowels—then gave him a warm bath, applied cups to the spine, and afterwards a blister to the same. After the bath, he seemed some better. Between that time and daylight I gave him about an ounce of paregoric. In the mean time the paroxysms were not so frequent, and he slept some.

29th. I had the room darkened early and kept quiet. At 6 o'clock, A. M., ordered four grs. of quinine every hour, with just enough paregoric to keep him dozing (which was not much, as he was very susceptible of its effects). The most nutritious diet admitted.

30th, 8 o'clock, A. M. I am informed that the paroxysms were not so frequent or severe yesterday as they were the day before; slept well last night and was not then conscious of spasms, but now having one every few minutes—pulse 110 per minute. Evacuated his bowels by enema; continued the quinine as yesterday. At this visit, I discovered a fluctuating tumor about the size of a hen's egg in the instep of the diseased foot, which was very painful on pressure. The fluid did not feel like pus. The swelling was fast leaving the foot on the application of poultices and tinct. of iodine. I now began to think that this toe was the cause of all the trouble; and as the foot was in a very unhealthy condition I did not like to remove it (the toe) without first advising with some older surgeon.

May 1st. Was met by Dr. Hayward, of Tuscaloosa, in consultation. After a thorough examination, he also concluded the toe was the cause of the tetanus, but thought it best not to remove it in the present condition of the foot. His symptoms were a little better to-day; pulse 100 per minute; spasms not quite so frequent. Dr. H. could see no reason for changing a practice which seemed beneficial, but looked upon his recovery as *very* doubtful. We ordered 10 grs. quinine and ¼ gr. morph. every two hours; an enema of castor oil and turpentine.

May 2d. 8 o'clock, A. M. Just as he was yesterday. I ordered 15 grs. quinine and ¼ gr. morphine every two hours. This was kept up all day and night.

May 3d, 8 o'clock, A. M. Complains of his head roaring; pulse 90 per minute; profuse diaphoresis; skin cool; a spasm about every 15 minutes—said he was very weak, and appetite not good. Ordered 10 grs. quinine and ¼ gr. morphine every three hours, and food *ad libitum.*

May 4th. Very deaf; had no spasm last night, but slept all the time, only when awaked to take his medicine—pulse 72, and weak; skin cool; profuse diaphoresis; appetite returning; has a spasm about every half an hour. I ordered 5 grs. of quinine, and ¼ gr. of morphine every two hours. In the afternoon he became partially blind from the effects of the quinine, and I omitted it for six hours, when this unpleasant symptom subsided.

May 5th. No spasm last night, but an occasional one through the day. Ordered quinine 4 grs. and ¼ gr. of morphine every three hours.

It is useless to give further details. Suffice it to say that his improvement was gradual and continuous, and in two weeks he had no symptom of tetanus remaining. I gave the quinine in diminished doses for a week after. The tumor in the instep broke, discharging a considerable quantity of thin, unhealthy pus. I dressed this and the toe with quinine, and put the boy on cod-liver oil and a little brandy three times a day. At this time he is fatter than I ever saw him, and his foot nearly well. I think his toe will also get well without an amputation.

I had just read the case reported by Dr. Pye in the New Orleans Medical Journal, and re-published in your Journal in March. I thought smaller doses of quinine than those he recommended would answer the same purpose; for, the absorbents acting very suddenly and powerfully might produce alarming, if not fatal results, after such large quantities had been administered.

